# Monitoring Energy Balance, Turbulent Flux Partitioning, Evapotranspiration and Biophysical Parameters of *Nopalea cochenillifera* (Cactaceae) in the Brazilian Semi-Arid Environment

**DOI:** 10.3390/plants12132562

**Published:** 2023-07-06

**Authors:** Alexandre Maniçoba da Rosa Ferraz Jardim, José Edson Florentino de Morais, Luciana Sandra Bastos de Souza, Carlos André Alves de Souza, George do Nascimento Araújo Júnior, Cléber Pereira Alves, Gabriel Ítalo Novaes da Silva, Renan Matheus Cordeiro Leite, Magna Soelma Beserra de Moura, João L. M. P. de Lima, Thieres George Freire da Silva

**Affiliations:** 1 Department of Agricultural Engineering, Federal Rural University of Pernambuco, Dom Manoel de Medeiros Avenue, s/n, Dois Irmãos, Recife 52171-900, Pernambuco, Brazil; george.araujojr@ufrpe.br (G.d.N.A.J.); cleber.pereira@ufrpe.br (C.P.A.); gabrielitalo.novaes@gmail.com (G.Í.N.d.S.); thieres.silva@ufrpe.br (T.G.F.d.S.); 2 Department of Biodiversity, Institute of Bioscience, São Paulo State University—UNESP, Av. 24A, 1515, Rio Claro 13506-900, São Paulo, Brazil; 3 Academic Unit of Serra Talhada, Federal Rural University of Pernambuco, Gregório Ferraz Nogueira Avenue, s/n, Serra Talhada 56909-535, Pernambuco, Brazil; joseedson50@hotmail.com (J.E.F.d.M.); luciana.sandra@ufrpe.br (L.S.B.d.S.); carlosandre08_@msn.com (C.A.A.d.S.); renanmatheuscl@gmail.com (R.M.C.L.); 4 Brazilian Agricultural Research Corporation, Embrapa Semiarid, Petrolina 56302-970, Pernambuco, Brazil; magna.moura@embrapa.br; 5 MARE—Marine and Environmental Sciences Centre, ARNET—Aquatic Research Network, Department of Civil Engineering, Faculty of Sciences and Technology, University of Coimbra, 3030-788 Coimbra, Portugal; plima@dec.uc.pt

**Keywords:** net radiation, biomass yield, water relations, abiotic stress, climate, cactus

## Abstract

The in-situ quantification of turbulent flux and evapotranspiration (ET) is necessary to monitor crop performance in stressful environments. Although cacti can withstand stressful conditions, plant responses and plant–environment interactions remain unclear. Hence, the objective of our study was to investigate the interannual and seasonal behaviour of components of the surface energy balance, environmental conditions, morphophysiological parameters, biomass yield and water relations in a crop of *Nopalea cochenillifera* in the semi-arid region of Brazil. The data were collected from a micrometeorological tower between 2015 and 2017. The results demonstrate that net radiation was significantly higher during the wet season. Latent heat flux was not significant between the wet season and dry season. During the dry-wet transition season in particular, sensible heat flux was higher than during the other seasons. We observed a large decline in soil heat flux during the wet season. There was no difference in ET during the wet or dry seasons; however, there was a 40% reduction during the dry-wet transition. The wet seasons and wet-dry transition showed the lowest Evaporative Stress Index. The plants showed high cladode water content and biomass during the evaluation period. In conclusion, these findings indicate high rates of growth, high biomass and a high cladode water content and explain the response of the cactus regarding energy partitioning and ET.

## 1. Introduction

The cactus *Nopalea cochenillifera* (L.) Salm-Dyck is an important plant used in animal diets in semi-arid environments, particularly in the driest areas of north-eastern Brazil and around the world [1,2,3]. This species of the Cactaceae family is known worldwide for its tolerance to abiotic stress and is able to survive in places with low annual rainfall (250–450 mm) [1,2,3]. Plants in this family that have crassulacean acid metabolism (CAM) are more resistant and have greater water use efficiency (WUE) compared to plants with a C3 or C4 photosynthetic pathway [4,5,6,7]. For instance, *N. cochenillifera*, besides the CAM pathway for CO_2_ uptake, has vegetative structures called cladodes (i.e., modified succulent stems). These structures help with drought tolerance and provide the animals with a high concentration of non-fibrous carbohydrates (~54.3%), water (~900 g kg^−1^ dry matter), minerals (especially calcium, potassium and phosphorus) and high total nutrient digestibility. On the other hand, cladodes have low levels of dry matter, crude protein (~5.4%), neutral detergent fibre (~24.8%), and acid detergent fibre [8,9,10].

Due to their high productive performance, several studies have been carried out with cacti (*Opuntia* spp. and *Nopalea* spp.) in a semi-arid environment, adopting management practices or evaluating the water deficit on the growth and productivity of the crop (e.g., [1,2,7,11,12]). However, few studies have been developed to evaluate the plant–environment interaction using the energy balance method, some with *Opuntia* spp. [13,14] and some with other species and ecosystems that include Cactaceae [15,16,17,18]. Consoli et al. [13] evaluated ecophysiological variables and energy balance in an irrigated crop of *O. ficus-indica* under a semi-arid climate in Sicily (Italy). They reported the importance of applying a method that detected changes in plant transpiration on a daily scale under wet and dry conditions. Pierini et al. [19] reported that the heat flux is high in vegetation that includes species of cactus (*O. spinisior* and *O. engelmannii*) in Tucson in the Sonoran Desert (Arizona).

In terrestrial ecosystems, energy and water exchange plays a fundamental role in hydrological control, climate phenomena and species survival [20,21,22]. To quantify the land-atmosphere fluxes, and understand this partition, the surface energy balance (SEB) predicts variations in turbulent fluxes and evapotranspiration (ET) from interaction of the soil–vegetation–atmosphere system [23,24,25]. In addition to being considered a fairly robust and valid method under semi-arid conditions, it can be applied to different vegetated surfaces (e.g., areas of forest and agricultural crops) and areas with small footprints (i.e., different fetch-to-height ratios) [26,27,28]. Among the methods used to determine turbulent flux, the Bowen ratio indirectly quantifies the latent heat flux (*LE*) using net radiation (*R_n_*), soil heat flux (*G*) and the air temperature and humidity gradients [29,30]. In addition to being a low-cost method compared to the eddy covariance and weighing lysimeter methods, the Bowen ratio method uses simple sensors and is preferred in ET studies in agricultural ecosystems [28,31,32].

On a global scale, part of the water from ecosystems is returned to the atmosphere by the ET [33]. Basically, two processes are involved in ET: evaporation—the phenomenon concerning the change in the liquid phase from water to vapour—and transpiration—the process of transferring water via the plant structures (e.g., stomata) to the atmosphere in the form of water vapour. The sum of these two physical phenomena, i.e., evapotranspiration, consumes more than half of the solar energy, with approximately 60% of the precipitation being transferred to the atmosphere by the *LE* [26,34,35].

ET is perhaps one of the most studied variables in arid and semi-arid environments, helping to explain the response of the vegetation to water availability. However, in dry landscapes, the available energy is high, and when the ET decreases due to water availability, there is an increase in the sensible heat flux (*H*) [24,26,33,35]. Within this context, these factors can trigger conditions of stress for some plant species and, when added to the excessive heat flux and high energy, may become more hostile.

In the field, hydrological conditions (i.e., wet and dry seasons) can cause changes in the energy fluxes and ET on different scales. In addition, the vegetation can modify the release of energy in response to its growth and phenology [26,36,37]. In this respect, using such information as vegetation indices (e.g., cladode area index, and Normalised Difference Vegetation Index) and growth indices (e.g., growth rate and phenophases) can be fundamental in understanding plant feedback to growing conditions.

Finally, although there are studies on monitoring mass and energy fluxes in dry forests and grasslands in a semi-arid environment [23,38,39,40,41], as far as we know, there are no studies that report information on growth stages, ecophysiology or surface energy balance in *N. cochenillifera*. Therefore, we hypothesised that (i) even under adverse stress conditions, the performance of the cactus for energy flux and evapotranspiration remains good; and (ii) its growth, water relations and water and nutrient use efficiency are maintained throughout each season. Furthermore, we believe that our results will provide new insights into CAM plants and their exchange of energy with the ecosystem. The objective of this study was to evaluate the interannual and seasonal behaviour of components of the surface energy balance, environmental conditions, morphophysiological parameters and water relations during the wet and dry seasons in a crop of *N. cochenillifera* in the semi-arid region of Brazil (municipality of Mirandiba).

## 2. Results

### 2.1. Surface Energy Balance

**Figure 1:**
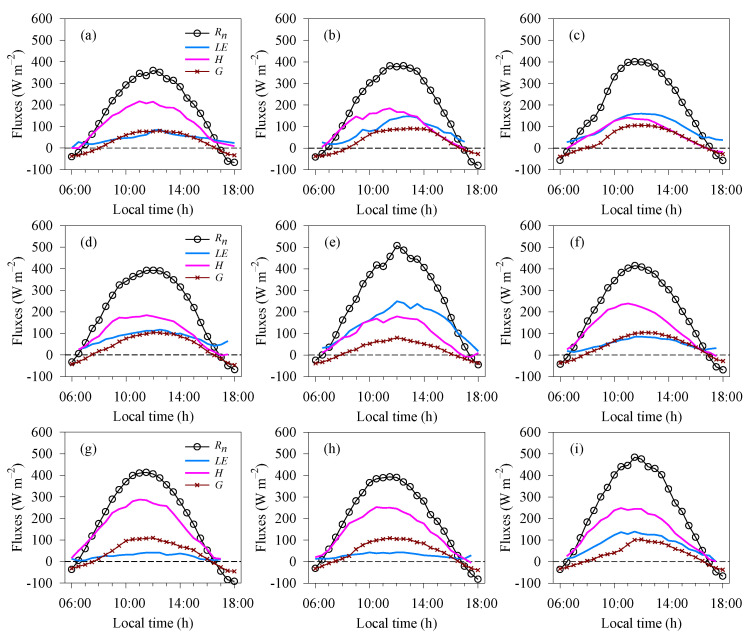
Hourly daytime mean energy flux over the area of cactus for different periods: wet season (**a**,**e**,**i**), wet-dry transition (**b**,**f**), dry season (**c**,**g**) and dry-wet transition (**d**,**h**). Here, *R_n_* is the net radiation, *LE* is the latent heat flux, *H* is the sensible heat flux and *G* is the soil heat flux, all calculated in W m^−2^. Note: panels (**a**–**c**) show the seasons for 2015; (**d**–**g**) for 2016; and (**h**,**i**) for 2017.

Figure 1 shows daytime values for the hourly variation in the energy balance fluxes during the experimental period, with the components being evaluated during the wet and dry seasons and their transitions (i.e., wet-dry and dry-wet seasons). During the wet season, the lowest daily values for net radiation (*R_n_*) were seen in 2015 (Figure 1a) and the maximum in 2016 (Figure 1e), with mean values of 172.21 and 247.92 W m^−2^, respectively. Among the energy fluxes, most of the *R_n_* came from the sensible heat flux (*H*), with the exception of 2015, which showed a mean value of 95.49 W m^−2^. In 2017, the highest value was seen for *H* (143.58 W m^−2^), whereas 2015 and 2016 saw the lowest and highest values for latent heat flux (*LE*), respectively, with mean values of 47.62 and 145.38 W m^−2^. During the wet season, the soil heat flux (*G*) was greater in 2017, showing a mean value of 32.13 W m^−2^, with a gradual increase throughout the day and maximum values around noon (11:00 to 14:00 local time). On the other hand, we found lower values for *G* during 2016, with a mean of 23.33 W m^−2^. As shown in Figure 1b,f (wet-dry transition), *R_n_* was 12% lower than during the wet season. There was a considerable variation in *LE* between 2015 and 2016, with mean values of 80.88 and 51.32 W m^−2^, respectively. This seasonal pattern was responsible for smaller turbulent fluxes; furthermore, there was an increase in *G* (34%) compared to the previous season.

The values for *R_n_* obtained throughout the day during the dry season in 2015 and 2016 (Figure 1c,g) were very similar, with mean values of 191.83 and 194.02 W m^−2^, respectively. These results for the energy budget were similar to the wet-dry transition season and different from those seen during the wet season. Specifically, *G* showed a significant increase (42%) compared to the wet season. For the turbulent fluxes, *LE* was 41% greater than *H* during the dry season in 2015. On the other hand, *H* showed contrasting behaviour in 2016 due to atmospheric conditions, being 141% higher than in 2015. Even so, the total turbulent flux was similar for the two years mentioned above, with a mean value of 176.87 W m^−2^. In general, the turbulent fluxes exhibited opposite trends over time and were the components with the greatest contribution to the energy budget. Figure 1d,h show the daily fluxes during the dry-wet transition season for 2016 and 2017. The result for *R_n_* in 2016 was 3.79% higher than in 2017, and comparing the two transition seasons, the value for *R_n_* during the dry-wet transition season was higher than during the wet-dry transition (4%). There was a large temporal variation in *G* during the study period. During the dry-wet transition season especially, *G* was approximately 18% higher than during the other seasons. Despite making a significant contribution to the energy balance, our results for *H* were approximately 2% (wet season), 6% (wet-dry transition) and 7% (dry season) higher during the dry-wet transition season. Furthermore, for the same season, which includes a dry phase, there was a reduction in *LE* (26%) compared to the other seasons. Based on this, it is clear that most of the available net surface energy contributed to the sensible heat flux.

**Figure 2:**
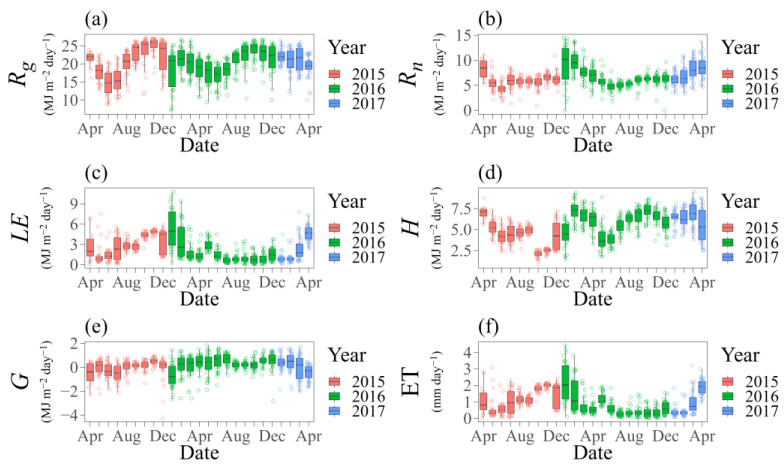
Time series for the energy budget in an area of cactus from 2015 to 2017. *R_g_* is the global solar radiation (**a**), *R_n_* is the net radiation (**b**), *LE* is the latent heat flux (**c**), *H* is the sensible heat flux (**d**), *G* is the soil heat flux (**e**) and ET is the evapotranspiration (**f**). The boxplots show the median; horizontal bars represent the 25th, 50th and 75th percentiles; whiskers (lower and upper) represent the 1.5× interquartile ranges. Corresponding data are represented by circles.

On a temporal scale, changes can clearly be seen in the energy budget throughout each year (Figure 2). The mean values for global solar radiation (*R_g_*) decreased significantly from May to July, being 22% lower in relation to the other years (Figure 2a). We also saw a greater range in December 2015 (median 24.38 MJ m^−2^ day^−1^, interquartile range 18.47 to 25.92) and January 2016 (median 21 MJ m^−2^ day^−1^, interquartile range 13.72 to 22.33). In 2015, 2016 and 2017, the mean values for *R_g_* were 20.45, 20.27 and 20.84 MJ m^−2^ day^−1^, respectively, despite an intermonthly variation (median 20.96 MJ m^−2^ day^−1^, interquartile range 17.75 to 23.73). Figure 2b shows the variation in *R_n_* throughout the experiment. In April 2015, January and February 2016 and from March to April 2017, the values for *R_n_* were higher, with a mean of 8.82 MJ m^−2^ day^−1^. However, the mean *R_n_* for the three years was 6.52 MJ m^−2^ day^−1^ (median 6.15, interquartile range 5.27 to 7.21), with a reduction of 26% compared to the months with the higher values shown above. As a result of cloudiness, *R_n_* had the lowest values in June, with a mean of 4.51 MJ m^−2^ day^−1^.

Figure 2c,d show the turbulent fluxes in the energy balance, with a mean value for *LE* of 2.32 MJ m^−2^ day^−1^ and maximum and minimum values of 5.41 and 0.70 MJ m^−2^ day^−1^, respectively, in January 2016 (median 4.97 MJ m^−2^ day^−1^, interquartile range 2.88 to 7.83) and July 2016 (median 0.56 MJ m^−2^ day^−1^, interquartile range 0.31 to 0.98). After the occurrence of high *LE* values from January to April 2016, there was a significant value (2.92 MJ m^−2^ day^−1^) in May 2016 due to the rainfall events. Obvious inversion of the turbulent fluxes can be seen from the values for *LE* in relation to *H* (Figure 2c,d). Although the values for *LE* were relatively high, the energy consumption was synchronous with that of *H*. During 2015–2017, *H* showed a mean value of 5.39 MJ m^−2^ day^−1^. Interestingly, the minimum values of *H*, with a mean of 2.11 MJ m^−2^ day^−1^ (median 2.21 MJ m^−2^ day^−1^, interquartile range 1.93 to 2.35), and the maximum values, with a mean of 7.21 MJ m^−2^ day^−1^ (median 7.24 MJ m^−2^ day^−1^, interquartile range 6.80 to 7.86), occurred in October 2015 and 2016, respectively, and due to the influence of the rainfall, resulted in a difference of 71%. Furthermore, *H* gradually increased in value from July to September 2015 and July to October 2016, with mean values of 4.70 and 6.37 MJ m^−2^ day^−1^ during this period, respectively.

Figure 2e,f show the behaviour of *G* and of the evapotranspiration (ET). For *G*, the values were more negative during 2015 (April to July, and December), as well as in January 2016 and April 2017. As shown, the minimum values (median –0.77 MJ m^−2^ day^−1^, interquartile range −1.37 to 0.10) and maximum values (median 0.71 MJ m^−2^ day^−1^, interquartile range 0.36 to 1.07) ranged, on average, from −0.65 to 0.67 MJ m^−2^ day^−1^, respectively. We found high values for ET when *G* and *H* were low. Despite the interannual variability, the range of values for ET agreed with the turbulent heat fluxes, ranging from 0.29 to 2.22 mm day^−1^ (median 0.23 MJ m^−2^ day^−1^, interquartile range 0.13 to 0.40 and median 2.03 MJ m^−2^ day^−1^, interquartile range 1.18 to 3.21, respectively), with a mean of 0.95 mm day^−1^ during the period under study.

**Figure 3:**
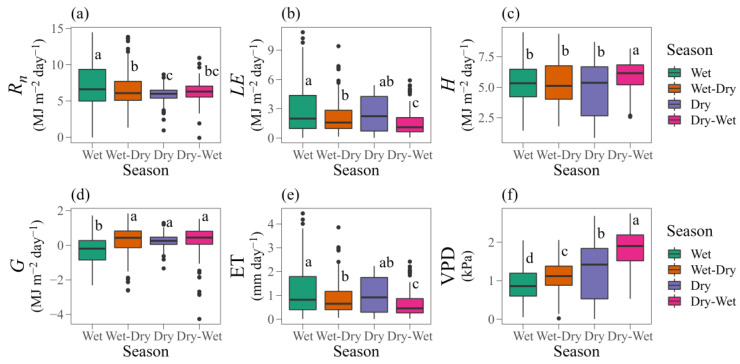
Boxplot of seasonal variations in energy and water exchange in an area of cactus. *R_n_* is the net radiation (**a**), *LE* is the latent heat flux (**b**), *H* is the sensible heat flux (**c**), *G* is the soil heat flux (**d**), ET is the evapotranspiration (**e**) and VPD is the vapour pressure deficit (**f**). The boxplots show the median; horizontal bars represent the 25th, 50th and 75th percentiles; whiskers (lower and upper) represent the 1.5× interquartile ranges; dots represent outliers. Significance was calculated using one-way analysis of variance (ANOVA) with Tukey’s honestly significant difference (HSD) post hoc test. Different letters above each box indicate significant differences (*p* < 0.05).

In Figure 3 and Table 1, we present a detailed seasonal boxplot analysis of the energy budget, heat exchange capacity and water vapour in the cactus during the four seasons (wet, wet-dry, dry, and dry-wet), together with the energy partition ratios. For *R_n_*, the values were significantly higher during the wet season (Tukey’s HSD test, *p* < 0.05; median 6.61 MJ m^−2^ day^−1^, interquartile range 4.99 to 9.37), being on average 14% higher than during the other seasons (Figure 3a). Despite showing similar mean values for *R_n_*, during the dry season, the energy was 7% lower than during the dry-wet transition season. For *LE* (Figure 3b), there was no significant difference (*p* > 0.05) between the wet and dry seasons, with mean values of 2.87 and 2.41 MJ m^−2^ day^−1^, respectively. On the other hand, there was a 25% reduction (*p* < 0.05) in *LE* with the arrival of the wet-dry transition season. Notably, the lowest and highest mean values for *LE* and *H*, 1.57 and 5.89 MJ m^−2^ day^−1^, respectively, were seen during the dry-wet transition season. These effects were even greater for *H* (Figure 3c), with significantly higher values (*p* < 0.05) during the dry-wet transition season (median = 6.16 MJ m^−2^ day^−1^, interquartile range 5.20 to 6.84). In general, during the dry-wet transition season, *H* was 13% greater than during the other seasons, showing that in the cactus under a semi-arid environment, most of the *R_n_* was destined for this process (Figure 3c). Verifying the energy partitions (Table 1), the *H*/*R_n_* ratio was generally responsible for the highest power consumption (58.5%). The *LE*/*R_n_* ratio varied between 17% and 30%, with a total mean value of 24% over all the seasons. In addition, the *G*/*R_n_* ratio reached an average of 17.5%, with the smallest partition (13%) during the wet season (Table 1).

The lowest values for *G* (Figure 3d) were seen during the wet season (−0.28 to 0.28 MJ m^−2^ day^−1^), with a marked reduction of 203% in relation to the other seasons. The sharp decrease in *G* and the *G*/*R_n_* ratio during the wet season (Table 1) is clearly due to the high volumes of rainfall and soil moisture during this period. Similar to the behaviour of *LE*, there was no significant difference in ET values during the wet or dry seasons (Figure 3e). This confirms the hypothesis that ET is maintained during the dry season and decreases during the dry-wet transition season (median 0.46 MJ m^−2^ day^−1^, interquartile range 0.26 to 0.86). We saw reductions of 40.31% and 26.68% during the dry-wet transition when comparing the wet and dry seasons and the wet-dry transition season, respectively (*p* < 0.05).

There was a clear significant seasonal variation in the vapour pressure deficit (VPD) (Figure 3f), with a mean of 1.82 kPa during the dry-wet transition season, being 45% and 97% higher during the dry season (median 1.42 kPa, interquartile range 0.53 to 1.84) and wet season (median 0.86 kPa, interquartile range 0.60 to 1.20), respectively. Furthermore, there was a significant difference (*p* < 0.05) when comparing the VPD of the transition seasons, where the VPD of the wet-dry season was 38% lower than that of dry-wet season (median 1.12 and 1.90 kPa, interquartile ranges 0.88 to 1.38 and 1.52 to 2.19, respectively).

### 2.2. NDVI Signatures, Environmental Data and the Impact of Drought

**Figure 4:**
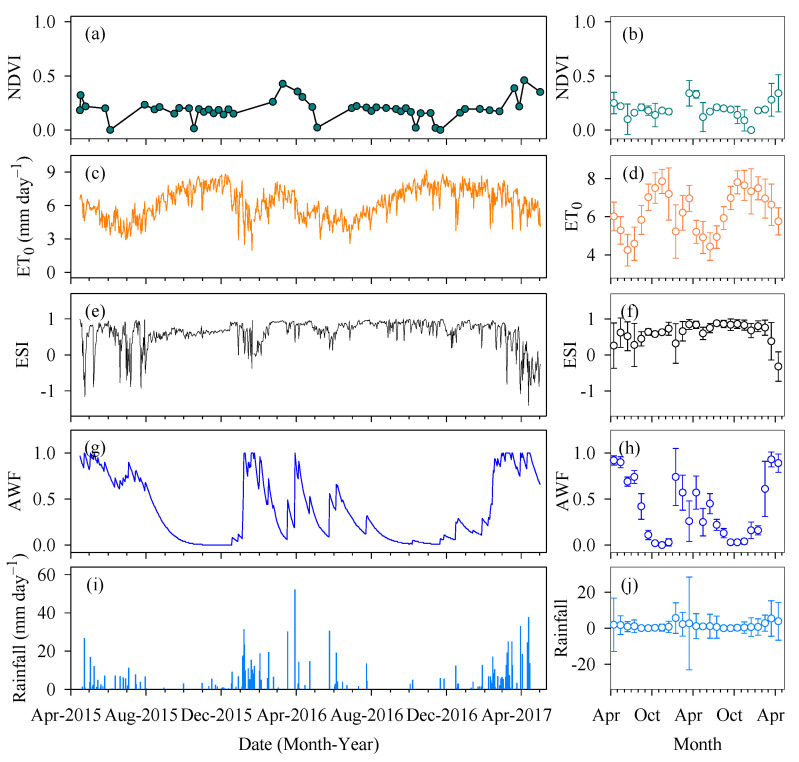
Temporal evolution of the Normalised Difference Vegetation Index [NDVI] (**a**), reference evapotranspiration [ET_0_] (**c**), Evaporative Stress Index [ESI] (**e**), available water fraction [AWF] (**g**) and rainfall (**i**) for an area cultivated with cactus. The five panels (**b**,**d**,**f**,**h**,**j**) show monthly results over each year for NDVI, ET_0_, ESI, AWF and rainfall during the experimental period, respectively. Data with error bars represent the mean ± SD (standard deviation).

**Figure 5:**
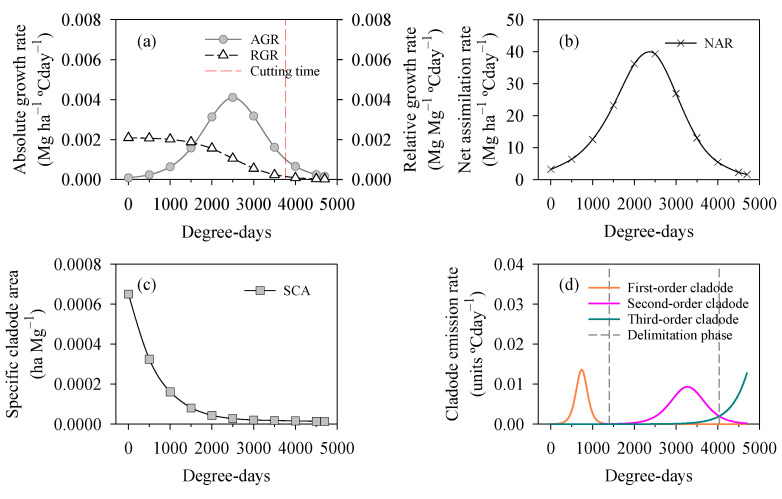
Absolute growth rate—AGR and relative growth rate—RGR (**a**), net assimilation rate—NAR (**b**), specific cladode area—SCA (**c**) and cladode emission rate (**d**) in *N. cochenillifera* (L.) Salm-Dyck under a semi-arid environment.

We selected a time series for the Normalised Difference Vegetation Index (NDVI), reference evapotranspiration (ET_0_), Evaporative Stress Index (ESI), available water fraction (AWF) and rainfall during the experimental period (Figure 4). There was no NDVI saturation during any of the cactus growing seasons (Figure 4a,b). The highest NDVI values were seen during the wet season, with a mean of 0.23. Every year, the mean values of the wet-dry, dry and dry-wet seasons (transitions) were, respectively, 9%, 26% and 39% lower than during the wet season. In addition, it should be noted that the NDVI decreased more significantly (17%) during the change from the dry season to the dry-wet transition season (Figure 4b). Despite oscillations in the NDVI trajectory in the area, the final periods of the cycle show greater consistency, with a rise in the index. Variations in the NDVI are influenced by the canopy cover, cladode morphometry (see Figure 5) and environmental conditions (Figure 4c,e). As shown in Figure 4c,d, the mean values for ET_0_ ranged from 4.26 (±0.83) to 7.85 (±0.63) mm day^−1^ (mean ± standard deviation), both variations occurring in 2015. Variations, both intermonthly and throughout the year, can also be seen in the ET_0_, with lower values between May and July, mainly due to the lower incidence of solar radiation and the lower air temperature (Figure 4c). During the present study, the ET_0_ was, on average, 6.24 mm day^−1^, with emphasis on the dry season (6.87 mm day^−1^) and the dry-wet transition season (6.78 mm day^−1^). On the other hand, during the wet and wet-dry seasons, we found lower values of 5.79 and 5.33 mm day^−1^, respectively. Although high ET_0_ values can be found for approximately 48% of the experimental period, the values remained below 6.24 mm day^−1^.

The monthly mean ESI ranged from −0.32 to 0.88 (Figure 4f). We saw negative ESI values (Figure 4e,f) due to the concentrated rainfall events, favouring greater soil moisture (Figure 4g,i). The wet season and the wet-dry transition season had the lowest ESI values, with means of 0.41 and 0.56, respectively. On the other hand, 2016 saw an exception in the behaviour of the ESI during the wet-dry transition season, with mean values greater than 0.7. During the dry season, the index was 35% higher than during the other seasons, reaching 78% compared to the wet season. This clearly confirms that the most stressful conditions occur during the dry season, which in turn has a high atmospheric demand. The variations in AWF largely occurred together with those of the above-mentioned variables (Figure 4g); in addition, the greatest mean value (0.77) was seen during the wet season. The monthly variation in AWF in the soil ranged from 0.002 to 0.931, and notably, these variations showed more critical values during the dry season, with means of 0.12 and 0.09 during 2015 and 2016, respectively. However, even with a water scarcity during the wet-dry and dry-wet transition seasons, the AWF was 0.48 and 0.31, respectively, being 289% greater compared to the dry season. Although the four seasons under study differ when compared to each other, there was a similar trend in their behaviour for the same season in each of the different years (Figure 4h).

We found a clear variation in rainfall events during the experimental period (Figure 4i,j). The values for 2015, 2016 and 2017 were 183.31 (±17.53), 477.13 (±49.19) and 382.78 (±61.71) mm year^−1^, respectively, a total of 1043.22 mm (347.74 ± 150.01 mm year^−1^). In 2016, the period with the greatest accumulation of rain, more than 50% of the rain was concentrated from January to February (wet season). In addition, it should be noted that in March of the same year, the rainfall events were less homogenous, resulting in higher values for the standard deviation. In general, the months from August to November show an extreme water deficit, with the monthly rainfall varying from 0 to 11.44 mm (Figure 4j).

### 2.3. Growth Parameters, Phenological Characteristics, and Cutting Time

The curves of the morphophysiological parameters, phenophases and cutting time of the cactus are shown in Figure 5. Analysing the absolute growth rate (AGR), we found that the cactus used more than 53% of the thermal time to reach the maximum AGR (0.0041 Mg ha^−1^ °Cday^−1^) at 2500 °Cday. Based on the AGR, the ideal cutting time of the plants was at 3760 °Cday, although the species was harvested at 4700 °Cday (Figure 5a). Notably, the relative growth rate (RGR) was higher during the initial growth period and gradually decreased until it reached minimum values. The maximum RGR value of the cactus was 0.0021 Mg Mg^−1^ °Cday^−1^. Again, both growth rates (i.e., AGR and RGR) showed a significant fall in biomass accumulation upon reaching the cutting time. When analysing the net assimilation rate (NAR), our results showed a mean NAR of 17.76 Mg ha^−1^ °Cday^−1^, with a variation of 1.60 to 40.22 Mg ha^−1^ °Cday^−1^. Although the NAR was higher at 2500 °Cday, there was a 50% reduction in the photosynthetic capacity of the cactus by the end of the cycle (4700 °Cday) compared to the initial growth period (Figure 5b).

Figure 5c,d show the performance of the specific cladode area (SCA) and cladode emission rate. The SCA values were higher at the start and decreased significantly over time, as was also seen with the RGR. The maximum SCA reached by the cactus was 0.00065 ha Mg^−1^; furthermore, when the plants reached the thermal sum of 1500 °Cday, there was a mean reduction of 0.00002 ha Mg^−1^ in SCA over time. Our results show that the cactus had three cladode phenophases (Figure 5d). The first-order cladodes reached the highest emission rate during these phenophases, with a mean of 0.0136 units °Cday. There were reductions of 32% and 5% in phenophases two and three, respectively, relative to the first phenophase. Interestingly, we also found a prolongation of phenophase two, with an accumulated thermal time of 2632 °Cday, while the first phenophase (1402 accumulated °Cday) and third phenophase (666 °Cday, with the lowest cumulative value) were shorter. We did not completely analyse the third phenophase, as the experiment had already been harvested before the phenophase ended.

### 2.4. Water Relations, Bowen Ratio, Biophysical Efficiency and Yield

**Figure 6:**
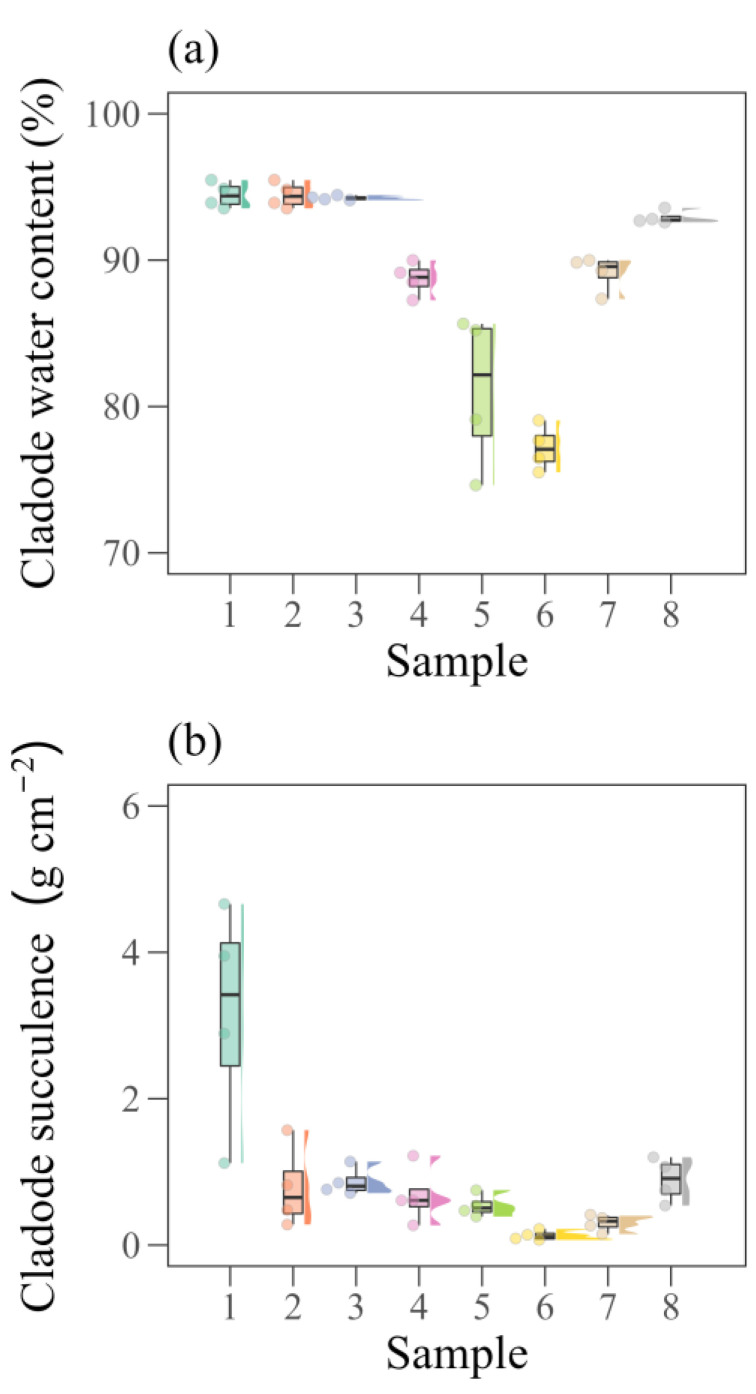
Cladode water content (**a**) and cladode succulence (**b**) in *N. cochenillifera* (L.) Salm-Dyck over time (2015 to 2017) under a semi-arid environment. The boxplots show the median; horizontal bars represent the 25th, 50th and 75th percentiles; whiskers (lower and upper) represent the 1.5× interquartile ranges. The colouring of each boxplot indicates the sampling period.

We found significant variation in the water relations of the cladodes throughout the experimental period (Figure 6). The cladode water content (CWC) had a mean value of 89.03%, varying from 77% to 95%, with interquartile ranges of 76% to 78% and 94% to 95%, respectively (Figure 6a). We also found a more-expressive gradual loss of cladode turgidity between samples four and six, and later, the rehydration of the cladodes. Samples five and six included October to December 2016, with low rainfall and low soil moisture (see Figure 4). Although the lower water availability caused significant dehydration, the cladodes rehydrated at the end of the cycle, with an increase of 20% in the CWC. From the second sample onwards, the plants showed a similar variation in cladode succulence to that of the CWC (Figure 6b). On the other hand, due to the high turgidity and smaller cladode area, succulence was generally greater in the first sample (3.16 g cm^−2^). The mean value for cladode succulence was 0.92 g cm^−2^, ranging from 0.07 to 4.66 g cm^−2^. The magnitude of the changes in water relations, whether increasing or decreasing, paralleled the levels of water availability and cladode development.

Table 2 shows our results for the efficiency of the biophysical parameters, the Bowen ratio and the biomass yield in the cactus at the end of the experimental period. The high radiation use efficiency (RUE) of the cactus can clearly be seen, with a mean value of 3.95 g MJ^−1^, which favoured the process of photosynthesis, and consequently, significant biomass conversion, with a mean of 12.47 Mg ha^−1^ dry matter. In addition, a value of 1.75 kg m^−3^ was found for water use efficiency (WUE). Considering the information provided by the surface energy distribution between the turbulent fluxes, our results point to a mean Bowen ratio (*β*) of 3.53 (Table 2).

According to our results, the cactus showed greater efficiency in the use of phosphorus and potassium, with mean values of 62.13 and 50.88 mg m^−3^, respectively (Table 2). Calcium use efficiency was 133% higher than that of magnesium. Clearly, the cactus was highly efficient in using the above nutrients, while sodium use efficiency was low (0.22 mg m^−3^), which indicates greater selectivity of the absorption channels for K^+^ in relation to Na^+^.

### 2.5. Interrelationships between Environmental Variables and Plant Responses

**Figure 7:**
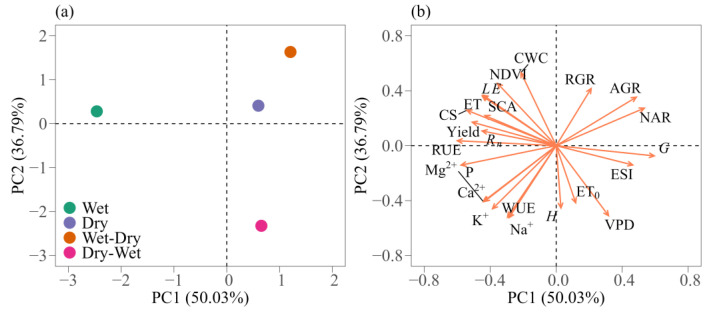
Principal component analysis (PCA) ordered biplot of environmental and plant factors. Score plot (**a**) and loading plot (**b**) of the first two principal components (PC) during the wet and dry seasons and transition periods. P (phosphorus), Mg^2+^ (magnesium), Ca^2+^ (calcium), K^+^ (potassium) and Na^+^ (sodium) refer to the efficiency of use of each nutrient. The following abbreviations are used: net radiation (*R_n_*), latent heat flux (*LE*), sensible heat flux (*H*), soil heat flux (*G*), evapotranspiration (ET), vapour pressure deficit (VPD), Evaporative Stress Index (ESI), Normalised Difference Vegetation Index (NDVI), water use efficiency (WUE), reference evapotranspiration (ET_0_), absolute growth rate (AGR), relative growth rate (RGR), net assimilation rate (NAR), specific cladode area (SCA), cladode water content (CWC), cladode succulence (CS), radiation use efficiency (RUE) and biomass yield (Yield).

The interrelationship between the environmental variables and the plant variables can be explained using principal component analysis (PCA) (Figure 7). Our results show that the first two principal components were responsible for 86.82% of the total variance, with a strong relationship between the environmental and plant variables during the growing seasons. Despite presenting an eigenvalue greater than 1, the third principal component was not shown due to the lack of any information relevant to the present study. On the other hand, with eigenvalues also greater than 1, the first principal component (PC1) contributed with 50.03%, while the second principal component (PC2) contributed with 36.79% of the total variance. The PCA revealed the clear separation of the growing seasons along the two dimensions of the principal component (Figure 7a). The wet and dry seasons and the wet-dry transition season presented the greatest contribution to PC1, with scores of −2.46, 0.60 and 1.21, respectively. For PC2, the two transition seasons contributed the most, with scores of −2.32 (dry-wet) and 1.63 (wet-dry). In addition to the higher PC1 scores, there was an obvious difference between the groups of environmental and plant variables.

The variables *G*, NAR, AGR, ESI, VPD, RGR, ET_0_ and *H* were positively correlated with PC1, with loadings varying from 0.59 to 0.03 (Figure 7b). On the other hand, the remaining variables (i.e., CWC, Na^+^, WUE, NDVI, K^+^, SCA, Ca^2+^, Mg^2+^, *LE*, ET, *R_n_*, Yield, CS, P, and RUE) showed a negative correlation with PC1, with loadings ranging from −0.21 to −0.60. The dry season and wet-dry transition showed the highest correlation (most-positive) with *G* (0.59), NAR (0.53), AGR (0.49) and RGR (0.21), with the first three having the greatest PC1 loadings. The results clearly show that for the above-mentioned seasons, the cactus shows lower nutrient and water use efficiency (i.e., most-negative PC scores). In particular, the group of variables showing a high correlation with the atmospheric conditions (i.e., *H*, ET_0_, VPD and ESI) did so during the dry-wet transition season. In addition, the wet season showed a positive correlation with the largest grouping of biophysical and plant variables (Figure 7b).

All the variables grouped together during the wet season (e.g., CWC, NDVI, *LE*, ET, CS, SCA, Yield, *R_n_* and RUE) had the highest (most positive) contribution to the total explained variance for PC2 (36.79%). The variables with the highest loading during the wet season were CWC (0.53) and NDVI (0.45). In addition, during the wet season, the cactus showed greater biomass yield, *LE* and ET than during the other seasons under study. Finally, the correlation between the variables and the wet season shows that the cactus had a greater SCA, CS and RUE, which explains the greater NDVI and biomass yield loadings.

## 3. Discussion

### 3.1. Mean Daytime Patterns, Seasonal Variations in the Energy Fluxes and Evapotranspiration

In semi-arid areas with a vegetated surface, the energy balance undergoes changes in the soil-atmosphere system [33]. The hourly energy partition showed the most-sensitive variations, with changes in the fluxes during the morning and afternoon (Figure 1). On the other hand, on a daily scale, the behaviour was similar but of different magnitudes over the years, as well as during the wet to dry and transition seasons (Figure 2). These more marked variations in turbulent flux and soil heat flux were also seen by Shao et al. [39] and require greater attention when there is excessive heating of the environment since this can compromise photosynthetic efficiency. Evaporation and transpiration can reduce the heat stored in the soil and in the plant; however, with the cactus being a CAM plant, transpiration is almost zero during the day [3,4,6], resulting in a smaller contribution to the *LE*.

Latent heat flux and sensible heat flux are the main variables in net energy consumption, with *H* prominent in the energy budget of a surface cultivated with cactus. In some cases, *LE* is less significant, an indication of environmental water limitations. Most of the time, daytime fluxes show a downward concave shape, with minimum values occurring at dawn and dusk [26,42]. It is possible that the proximity of turbulent fluxes in the morning (before 10:00) during some wet and dry seasons may have been due to the increased atmospheric demand and soil moisture that favour evapotranspiration. CAM plants have the ability to cool the soil overnight [43], and although their metabolism is generally nocturnal, we believe that this thermal reduction lasted until the early hours of the morning [27]. In this case, evaporation from the soil may have resulted in greater change in latent heat flux. Unlike forest species, cacti do not store intercepted water in the canopy but in the parenchymatic tissue. Such plants are able to carry out hydraulic redistribution of the water in the soil, making it available in the surface layers. In this way, the evaporated water may come mostly from the soil [44,45].

Based on the above analyses, we found similarities in the *R_n_* curves (Figure 1). However, it is clear that *R_n_* was significantly higher during the wet season due to the greater absorption of thermal and radiant energy by the humid atmosphere (Figure 3), contributing to the *LE* [23,40]. Using the surface energy balance with the cactus *O. ficus-indica* (L.) Mill., Consoli et al. [13] also found a higher *R_n_* (~13 MJ m^−2^ day^−1^) in a semi-arid environment. The turbulent fluxes and soil heat flux showed marked variations on an hourly scale and during the seasons under study (Figure 1 and Figure 2). *G* was clearly more expressive during the afternoon, mainly due to the accumulation of energy throughout the morning, and with the greater soil moisture and radiation, there was an increase in the thermal conductivity of the soil [33]. In addition, the lower values for *G* may be associated with the biophysical and growth parameters of the plants [46]. To substantiate this further, the cactus showed a high growth rate and high NDVI even when there was a reduction in soil moisture, resulting in reduced radiation input due to the greater soil cover. Although the cactus does not present a denser, more-uniform canopy, the cladodes remain on the plant throughout the cycle, which may cause variations in energy exchange due to the spaces between them. With greater results than those reported in the present study, Flanagan and Flanagan [47] found a mean value of 2 MJ m^−2^ day^−1^ for *G* in an area of saguaro cactus (*Carnegiea gigantea*). Furthermore, the authors point out that the greatest net energy dissipation was via surface heating and the sensible heat flux. Under semi-arid conditions and vegetation consisting of the cacti *O. spinisior* and *O. engelmannii*, Pierini et al. [19] also found higher values for the sensible heat flux compared to the latent heat flux.

Indeed, meteorological conditions have a strong relationship with the variables of the surface energy balance (Figure 2 and Table 1). We saw a similar strong consistency in global solar radiation (*R_g_*), with seasonal trends consistent with the climate in the region [48,49]. Areas of low latitude and dry climate in particular have a lower *LE* and greater *H* [50]. This is because the incidence of solar radiation is high, and due to the low water availability, the latent heat flux is lower, resulting in limitations on evapotranspiration. The energy balance partition patterns were consistent with the other environmental variables. For example, when the sensible heat energy was low, the cactus increased evapotranspiration, behaving as an important energy sink (Figure 2). Other studies point to variations in turbulent fluxes in areas of Cactaceae and the effects of changes in the energy balance due to water availability (e.g., [13,14,37,47]).

The vapour pressure deficit (VPD) is closely related to ET [51]. Even under deficit conditions, more specifically during the dry season, the cactus maintained ET on a significant scale. Interestingly, even during extremely critical periods (e.g., during the dry season and dry-wet transition), the cactus can keep water lost to the atmosphere well below that seen in species with C3 and C4 metabolism. For these plants, maintaining transpiration during critical periods has important benefits, such as the removal of excessive heat from the cladodes and the maintenance of net photosynthesis [14]. This supports our hypothesis that the cactus manages to maintain satisfactory ET. Under rainfed conditions, as in the present study, the mean was 0.95 mm day^−1^ during the experimental period, with a value of 1.18 mm day^−1^ during the wet season, 0.99 mm day^−1^ during the dry season, 0.88 mm day^−1^ during the wet-dry transition and 0.65 mm day^−1^ during the dry-wet transition (Figure 3). In contrast, studies by Consoli et al. [13], Goldstein et al. [52] and Lima et al. [53], evaluating species of cactus (*Opuntia* spp.) under irrigated conditions, found values for ET greater than 2 mm day^−1^. Under rainfed conditions, Han and Felker [54] found a mean daily value of 1.44 mm in *O. ellisiana*, while in a semi-arid ecosystem with cactus (*O. engelmannii*) and shrubs, Anderson and Vivoni [24] found approximate ET values ranging from 0 to 3.5 mm day^−1^.

### 3.2. Variations in the NDVI, Seasonal Environmental Changes and Soil Moisture

Vegetation and terrestrial ecosystems have intrinsic characteristics regarding environmental conditions and, consequently, different spectral responses. To evaluate plant behaviour, the Normalised Difference Vegetation Index (NDVI) is a very promising method that is widely used in arid and semi-arid ecosystems. This is because information in the near-infrared spectrum has a direct relationship with the photosynthetic rate of the vegetation and helps in estimating biomass accumulation [36]. For the cactus, we found an NDVI with a mean value of 0.19 throughout the experimental period (Figure 4). In addition, there are reports of a low NDVI and low vegetation indices in areas of cacti [55]. Similarly, Silva et al. [56] found vegetation indices ranging from 0.1 to 0.5 in areas cultivated with *O. stricta* and *N. cochenillifera* in the Agreste region of Pernambuco, Brazil. The quick response of the NDVI for cacti, even under low water availability, may be related to the roots that remain in the surface layers of the soil absorbing rainwater and water vapour from the air [55], thereby maintaining both growth and photosynthesis, even if at a reduced rate. Furthermore, if the high atmospheric demand is combined with water availability in the soil, plant evapotranspiration will be high. Variations in atmospheric demand are generally caused by water availability and the time of year. With a mean of 6.24 mm day^−1^ (Figure 4c), the results for reference evapotranspiration (ET_0_) were consistent with the environmental conditions and the rainfall events that occurred. The ET_0_ in semi-arid regions of Brazil is high due to the high incidence of radiation and low rainfall volumes and can vary on average from 6 mm day^−1^ in areas of cactus to more than 8 mm day^−1^ in a seasonally dry forest (Caatinga) [38,57].

In the case of environments with high atmospheric demand and low soil moisture, the Evaporative Stress Index (ESI) can identify problems in plant performance [58]. Although the ESI presented values close to one during the present study, with this value classified as a stressor for the crop [58], the cactus maintained its active development. The resistance to hostile climate and environmental factors is overcome due to the anatomical and morphophysiological characteristics of the plant [1,14,53,54]. On the other hand, when the periods of drought are more severe and prolonged, the yield, growth and biochemical and physiological parameters of the cactus may be impaired [1,7,59]. In addition, characteristics of the root system, parenchymal tissue and stomatal control allow the cactus to take advantage of the soil moisture and show good performance even when water availability is low [7]. The mean value of the rainfall was 347.74 mm year^−1^, within the values for plant survival [2,12].

### 3.3. Allometry, Phenological Phase, and Cutting Time

One characteristic of plants is to present differing adaptations, phenotypic plasticity and behaviour in terms of growth rate. Earlier studies (e.g., [11,60,61,62]) also reported similarities in the growth curves of the cactus; however, compared to genus *Opuntia*, genus *Nopalea* does not show very high rates of growth. When evaluating growth rates (i.e., absolute growth rate—AGR and relative growth rate—RGR) in *N. cochenillifera* under rainfed conditions in a semi-arid environment, Araújo Júnior et al. [11] found a lower AGR (<0.004 Mg ha^−1^ °Cday^−1^) and higher RGR (>0.002 Mg Mg^−1^ °Cday^−1^) compared to our results (Figure 5). The AGR helps explain the ratio of dry matter accumulation in the crop, while the RGR shows the relative increase in dry matter per unit of time [61]. Generally, a reduction in both of the above rates can clearly be seen throughout the cycle. This is because as plants grow, they consume photoassimilates, causing a natural reduction in these rates [11,60,61,62]. The ideal cutting time favours a more promising crop, with better use of photoassimilates and better biomass accumulation [61]. Other studies point out that the cactus shows variations in harvest time depending on the agronomic management and/or environmental conditions [11,60,61,62].

Our results showed high values for the cactus in terms of photosynthetic capacity, a ratio predicted by the net assimilation rate (NAR). From our findings, the crop presented significant net photosynthesis, with little influence from self-shading of the cladodes due to the smaller specific cladode area (SCA), favouring a high NAR (Figure 5b,c). For Scalisi et al. [7], morphometric variables such as cladode area help us understand the growth behaviour of plants. The number of cladodes is also a fundamental variable for understanding the phenology of the cactus. In the present study, the cactus presented up to the third phase, i.e., the successive emission of third-order cladodes (Figure 5d). This agrees with other studies on cactus subjected to a semi-arid environment [11,61]. These findings also confirm the hypothesis that plants continue to develop, increasing their growth rate under different environmental conditions. On the other hand, it is possible that, despite the plants having reached the third phase, the phases were prolonged due to the low water availability.

### 3.4. Water Relations and Biophysical Parameters of Cladodes

Anatomically, the cactus has the capacity to store water in various structures, such as parenchyma and hydrenchyma cells [1,7]. Our results showed that both the cladode water content and cladode succulence presented significant variations (Figure 6). During dry periods, samples five and six showed greater water loss; plants from sample six in particular lost more water due to the long period of drought [7]. In addition, when plants lose water to the environment, they reduce their cladode area, resulting in a loss of cell turgor and succulence (Figure 6b) [6,7,44]. Even with a significant loss of cladode water content, succulent plants are able to store water more efficiently than plants with a C3 or C4 photosynthetic pathway, favouring species survival and the metabolic pathways [6,59]. While the cladodes are still young and have a smaller area than mature cladodes, cladode succulence may be greater since the area is reduced and the water content is high, as seen in the first batch of samples (Figure 6b). These results corroborate those of Scalisi et al. [7], who found variations of approximately 45% to 85% in cladode water content. When the number of cladodes increases, their water content may decrease due to competition for water by the cladodes. Other studies point to variations of 60% to 95% in the cladode water content, influenced by soil moisture [63,64]. Such variations in turgor may occur due to the drought tolerance of the plant since the cactus is able to maintain turgor pressure for months [6,52].

When plants are highly efficient in using biophysical resources, the probability of achieving better yields is high [57]. The results shown here generally underline the high efficiency of the cactus in using radiation, water and nutrients (see Table 1). One of the factors to help in radiation use efficiency (RUE) is the canopy architecture, which favours the interception of photosynthetically active radiation (PAR) and the capture of CO_2_, to be later converted into biomass [6,47,65]. The relationship between RUE, water use efficiency (WUE) and nutrient use efficiency (NUE) is true since the plants showed high dry biomass yield. The study by Han and Felker [54] reported the high WUE of *O. ellisiana* (1 kg of dry matter per 162 kg of water); however, such results are still in the early stages for *N. cochenillifera* under rainfed conditions. Our findings of 1.75 kg m^−3^ are higher than those reported by Mbava et al. [66] for C3 and C4 species, e.g., wheat (1.18 kg m^−3^), sorghum (1.48 kg m^−3^), maize (1.47 kg m^−3^) and cotton (0.22 kg m^−3^).

As seen in earlier studies with the cactus [67,68,69], we found that the plants showed greater efficiency and accumulation for such nutrients as P, K^+^, Ca^2+^, Mg^2+^ and Na^+^ (Table 2), in order of use efficiency. Nedjimi [68] and Saraiva et al. [69], respectively evaluating *Opuntia* and *Nopalea*, also found greater concentrations of P and K^+^ in the cladodes. They concluded that cacti have higher concentrations of the above nutrients, favouring the nutritional value for human/animal consumption. In addition, the high concentration of nutrients such as K^+^ can help improve performance against abiotic factors, such as environmental changes, salinity and heat [1]. The high Bowen ratio was due to the greater heat flux (Table 2), conditions characteristic of a deficient climate [23], which can be damaging to crops. High values can also be seen for the *H*/*LE* ratio in an arid ecosystem of cactus [47]. The Bowen ratio can change depending on the meteorological variables, soil conditions and vegetation, in which case, adapted plants tend to suffer less. Despite the semi-arid conditions to which the plants were exposed, the cactus showed a high yield of dry biomass (mean of 12.47 Mg ha^−1^). In this respect, our data are superior to those found by Jardim et al. [1,57], where the cactus was harvested at a younger age in a semi-arid environment.

### 3.5. Principal Component Analysis (PCA)

For the purposes of this study, we applied PCA to the variables that were most influenced by the wet and dry seasons and their transitions (Figure 7). The analysis allowed a reduction in the data set, transforming the data into a series of interrelated variables without losing the principal characteristics of the data. Several variables were correlated with the growing seasons, and the first two principal components (PC) explained 86.82% of the total variance, which was distributed over the 23 variables of the PC1 and PC2 coordinates. The formed groups are clearly correlated based on water availability and the environmental conditions. This is because, in addition to the microclimate caused by the rainfall, according to Campos et al. [12], the cactus responds to different levels of soil water availability. Jardim et al. [1] used the PCA method to understand which environmental and plant variables have greater interaction with different cactus genotypes (*Opuntia* spp. and *Nopalea* spp.) irrigated with saline water in the semi-arid region of Brazil. Furthermore, according to the authors, this type of analysis helps in understanding how plants behave in the face of environmental stressors.

Previous studies showed the grouping of cladode and plant variables [1,2], where correlated variables of the same sign explain, for example, the greater yield of the plants. It is interesting to see water use efficiency grouped with nutrient use efficiency since these variables were inversely related to the growth rates, which explains their lesser accumulation during the wet-dry transition and dry season. Recently, several studies have identified variations in the energy budget during wet and dry seasons [22,23,33,51]. Plant evapotranspiration may be limited by the available energy, water and evaporative demand; thus, as the plants’ increased evapotranspiration, the heat stress index and evaporative demand were reduced. The information presented here implies a clear effect from the wet and dry seasons on energy balance, turbulent fluxes and plant responses due to the grouping of the variables and the contribution of each component.

## 4. Materials and Methods

### 4.1. Location and Information of the Experimental Area

**Figure 8:**
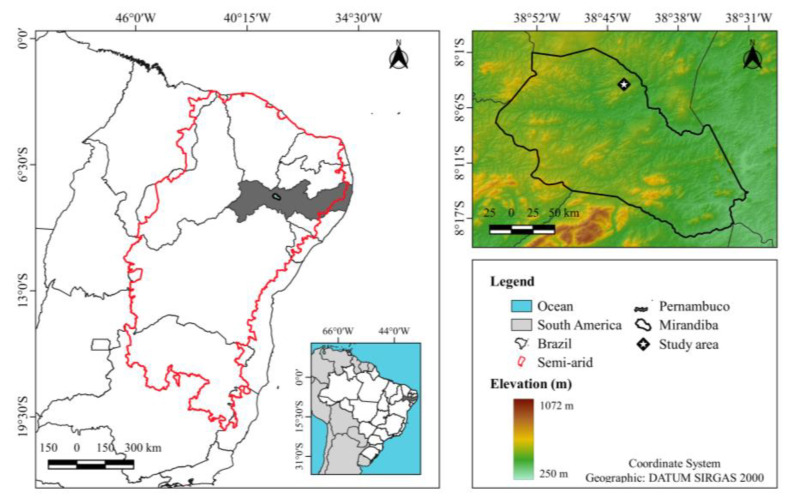
Geographic location of the study area in the district of Mirandiba, Pernambuco, Brazil. UTM projection (zone 24S), with the SIRGAS2000 Datum.

The experiment was conducted in a study area located in the district of Mirandiba, in the state of Pernambuco, Brazil (8°3.73′ S, 38°43.69′ W, altitude 490 m) under the conditions of a water deficit (Figure 8). According to the Köppen classification, the climate in the region is classified as semi-arid, type BSh (i.e., dry and hot with a rainy season in the summer) [70,71]. Rainfall predominates from January to June, with an annual average of 431.8 mm, average air temperature of 25.2 °C, relative humidity of 64.6%, and a high atmospheric demand that can reach 1600 mm year^−1^. The environment is characterised by high luminosity, irregular spatiotemporal distribution of the rainfall and the occurrence of periodic droughts.

Based on the beginning and end of the rainfall regime, we considered four seasons (wet, wet-dry transition, dry and dry-wet transition) for each year under study from 2015 to 2017. If the sum of rainfall values in the 30 days preceding or following the day under analysis is less than 20 mm, and there have been less than five rainy days, it is considered a dry season. However, if in the same time interval, rainfall values greater than 20 mm are recorded on five or more days, the season is considered wet. A transition season is determined when none of these criteria apply. If this happens after a dry season, it is said to be a dry-wet transition season, and when it occurs after a wet season, it is considered a wet-dry transition season [72,73]. For example, using this approach, the wet season included 21 February 2015 to 23 July 2015, 7 January 2016 to 1 February 2016 and 21 February 2017 to 1 July 2017; the wet-dry transition included 24 July 2015 to 21 August 2015 and 2 February 2016 to 26 July 2016; the dry season included 22 August 2015 to 7 December 2015 and 27 July 2016 to 17 November 2016 and the dry-wet transition included 8 December 2015 to 6 January 2016 and 18 November 2016 to 20 February 2017.

The soil in the experimental area was classified as a Chromic Luvisol [74], comprising 54% sand, 21% silt and 25% clay. Soil samples were collected at depths of 0–0.20 m, with a bulk density of 1.45 g cm^−3^ (measured by the cutting-ring sampling method), soil organic carbon content of 10.1 g kg^−1^, field capacity of 0.26 cm^3^ cm^−3^, soil pH of 6.1 (measured at a soil to water ratio of 1:2.5 using an ion pH meter), and terrain slope between 3% to 5%.

The experiment was conducted from April 2015 to April 2017 over a total area of 1.8 ha, using the cactus, *Nopalea cochenillifera* (L.) Salm-Dyck, a species tolerant to *Dactylopius opuntiae* Cockerell (Hemiptera: Dactylopiidae). The crop was planted in January 2015. After the initial soil preparation (i.e., ploughing, harrowing and furrowing), the cladodes were planted, leaving 50% of their height in the soil. The plants were arranged in single crop rows with the cladodes aligned bilaterally (i.e., parallel to each other), at a spacing of 2.0 × 0.50 m (10,000 plants ha^−1^). Cropping treatments (i.e., hand weeding and the application of herbicide and insecticide) were carried out whenever necessary to avoid competition with spontaneous plants and promote full growth of the crop. The cactus was grown under rainfed conditions throughout the evaluation cycle.

### 4.2. Measuring the Meteorological Variables

A 3-metre micrometeorological tower was installed in the centre of the experimental area. Net radiation (*R_n_*) data were measured using a closed-cell thermopile sensor (NR-Lite, Kipp and Zonen, Delft, Netherlands, accuracy = ±10 μV W^−1^ m^−2^). The soil heat flux (*G*) was measured at a depth of 0.05 m from the surface using a heat flux plate model HFT-3 (REBS, Hukseflux, Delft, Netherlands, accuracy = ±5% of reading) buried close to the crop row. Air temperature (*T_a_*) and relative humidity (*RH*) were determined using two aspiration psychrometers (height of 0.5 and 1.5 m above the surface of the soil). Wind speed and direction were measured using a Wind Sentry model 03002 anemometer (R. M. Young Company, Traverse City, MI, USA, accuracy = ±0.5 m s^−1^). Rainfall data were quantified using an automatic rain gauge (CS700-L, Hydrological Services Rain Gauge, Liverpool, Australia) installed 3 m above the canopy, and the photosynthetically active radiation (PAR) was measured using a point quantum sensor (LI-190SB, LI-COR Inc., Lincoln, NE, USA, accuracy = ±5% of reading) installed above and a 1 m line-quantum sensor (LI-191R, LI-COR, accuracy = ±5% of reading) below the cactus canopy. In addition, the height of the sensors met the fetch-to-height ratio of 100:1 [75].

In our study, the data were measured every 60 s by a CR10X data logger (Campbell Scientific Inc., North Logan, UT, USA) with a storage interval of 10 min. Measurements were collected continuously both day and night. However, the flux data used in applying the Bowen ratio-energy balance (BREB) method were considered on a daytime scale only. This is because at night, when the temperature/humidity gradients are small, erroneous flux data may be quantified. Each dataset is referred to in terms of local time (GMT-3).

### 4.3. Surface Energy Balance Method

We used the surface energy balance (SEB) method, which is based on the law of conservation of energy (Equation (1)).

(1) $$R_{n}\;-\;G=\mathrm{LE}+H\;$$

where *R_n_* is the net radiation (W m^−2^), *G* is the soil heat flux (W m^−2^), *LE* is the latent heat flux (W m^−2^) and *H* is the sensible heat flux (W m^−2^).

The turbulent flux components, i.e., sensible heat and latent heat fluxes were determined based on the Bowen ratio (*β*) (Equation (2)). The Bowen ratio method is widely used to partition energy flux components in relation to the total available energy (*R_n_* − *G*). We can therefore estimate this ratio by quantifying the temperature gradient and vapour pressure above the canopy [29]. In the present study, we assume similarity of equality between the turbulent transfer coefficients of the sensible heat (*K*_h_) or of the water vapour (*K*_w_) [76]. Therefore, latent heat flux (*LE*) and sensible heat flux (*H*) were estimated by combining the available energy balance and Bowen ratio (Equations (3) and (4), respectively).

(2) $$\;\beta =\frac{H}{\mathrm{LE}}=\left(\frac{P_{a}{\;\times \;c}_{p}}{\lambda \;\times \;\varepsilon }\times \frac{∆T}{∆e}\times \frac{K_{h}}{K_{w}}\right)$$

(3) $$\mathrm{LE}=\frac{R_{n}\;-\;G}{1+\beta }\;$$

(4) $$\;H=\frac{\beta }{1+\beta }\times \left(R_{n}\;-\;G\right)$$

where *β* is the Bowen ratio (dimensionless), *P_a_* is the atmospheric pressure (kPa), *c_p_* is the specific heat capacity of the air (1004.67 J kg^−1^ °C^−1^), *λ* is the latent heat of vaporisation (2.454 MJ kg^−1^ at 20 °C), *ε* is the ratio of the molecular weights of the air and water vapour (0.622), Δ*T* is the difference in air temperature between the two heights (°C), Δ*e* is the difference in vapour pressure between the two heights (kPa), *K*_h_ is the eddy diffusivity for heat (m^2^ s^−1^) and *K*_w_ is the eddy diffusivity for water vapour (m^2^ s^−1^). Quality control was carried out on the calculated *LE* as per Perez et al. [77].

Furthermore, in the present study, to calculate the energy balance closure, we ignored the energy from metabolic activities and heat storage in the plant tissue and in the canopy, as well as horizontal advection. This condition can be applied when the surface is uniform, keeping in mind that the vertical gradient that comprises the meteorological elements is far greater than the horizontal gradient. As such, these terms are ignored in building Equation (1) [30,31].

#### Data Selection Criteria for the Energy Balance Method

In the present study, for the acceptance and/or rejection criteria of the data collected by the Bowen ratio-energy balance (BREB) method, we used the approach proposed by Perez et al. [77] (Table 3). That said, the presence of abnormal data occurs when the available-energy heat flux (*R_n_* − *G*) is very small. In this way, faults may occur in applying the energy balance by the Bowen ratio (*β*) and later trigger several errors, e.g., when (1) the sensor resolution is inadequate to solve the gradient in Δ*T* and Δ*e*; (2) stable atmospheric conditions, e.g., at dawn and dusk, return *β* values close to −1, resulting in evapotranspiration tending to infinity, which is inconsistent; and (3) the conditions change abruptly, causing measurement errors (Table 4) [30,77,78,79].

### 4.4. Resource Use Efficiency

#### 4.4.1. Water Efficiency

The actual evapotranspiration component (ET, mm day^−1^) was calculated as described in Equation (5) [80]. To help understand the aboveground dry biomass yield in relation to the volume of water consumed, we calculated the water use efficiency (WUE, kg m^−3^) of the crop (Equation (6)) [81].

(5) $$\mathrm{ET}=86,400\;\times \frac{\mathrm{LE}}{\lambda {\;\times \;\rho }_{w}}$$

(6) $$\mathrm{WUE}=\frac{Y}{\mathrm{ET}}\;$$

where 86,400 is the time unit conversion factor (i.e., converting from seconds to days), *LE* is the latent heat flux measured over 24 h (W m^−2^), *ρ_w_* is the density of water (kg m^−3^), *Y* is the dry matter yield (kg ha^−1^) and ET is the total amount of seasonal evapotranspiration (m^3^ ha^−1^).

#### 4.4.2. Radiation Use Efficiency

The radiation use efficiency (RUE) of the cactus was calculated to describe the light-absorption dynamics of the crop (Equation (7)). To achieve this, we used the ratio between the total aboveground dry weight of the plant and the radiation intercepted throughout the growing season [82].

(7) $$\mathrm{RUE}=\frac{Y}{I_{0}\;\times \;f\mathrm{PAR}}$$

where RUE is the radiation use efficiency (g MJ^−1^), *I*_0_ is the amount of daily incident photosynthetically active radiation (PAR) above the canopy (MJ m^−2^) and *f*PAR is the fraction of intercepted photosynthetically active radiation.

The intercepted photosynthetically active radiation was estimated monthly using the AccuPAR LP-80 ceptometer (Decagon Devices, Logan, UT, USA), manually calibrated prior to taking the readings. To maintain consistent measurement conditions and avoid the influence of the angle of the sun’s rays, readings were taken under clear skies between 11:00 and 13:00, recording the incident radiation above and below the canopy. Three simultaneous readings were taken below the canopy (with the sensor rod positioned parallel and perpendicular to the crop rows) and one above, on four similar plants in the experimental area. We then calculated *f*PAR using Equation (8), and the light extinction coefficient (*k*) based on the Beer–Lambert law (Equation (9)). As the cactus has cladodes instead of leaves, we calculated *k* considering the cladode area index (CAI).

(8) $$\;f\mathrm{PAR}=\left(1\;-\;\frac{I_{t}}{I_{0}}\right)$$

(9) $$f\mathrm{PAR}=1\;-e^{\left(-k\;\times \;\mathrm{CAI}\right)}\;$$

where *I_t_* is the PAR measured at the bottom of the canopy and CAI is the cladode area index, determined according to Pinheiro et al. [83].

#### 4.4.3. Nutrient Use Efficiency

After cutting (aboveground biomass), the cladodes were weighed on an electronic scale to quantify the fresh biomass (g FM plant^−1^) and then dried in a forced air circulation oven at 55 °C to constant weight, i.e., dry matter per plant (g DM plant^−1^). The dried cladode samples were then ground using a Model 4 Wiley mill (Thomas Scientific, Swedesboro, NJ, USA) with a 1 mm sieve. The mineral-element concentration in the plant tissue was then determined: phosphorus (P, mg kg^−1^) using the vanadate-molybdate method with readings by UV-visible spectrophotometry at 430 nm [67]; potassium (K^+^, mg kg^−1^) and sodium (Na^+^, mg kg^−1^) by flame photometry [84]; and Calcium (Ca^2+^, mg kg^−1^) and magnesium (Mg^2+^, mg kg^−1^) by atomic absorption spectrophotometry [85]. Finally, nutrient use efficiency (NUE, mg m^−3^) was calculated as per Equation (10).

(10) $$\mathrm{NUE}=\frac{Y\;\times \;\mathrm{Nu}}{\mathrm{ET}}$$

where *Nu* is the concentration of the nutrient in the analysed sample of plant tissue (mg kg^−1^). To improve understanding of nutrient uptake by the plants, we adapted Equation (10) [86], making it a function of crop evapotranspiration. This adaptation affords more clarity in explaining the nutrient uptake capacity of the plant from the soil solution, together with the water consumption lost through evapotranspiration.

### 4.5. Analysing Growth, Phenology, Cutting Time and Yield

Morphometric data were collected monthly, and samples of plant biomass were taken at 60-day intervals. Four plants were measured for each analysis in each evaluation period. Height was evaluated considering the vertical distance from the ground to the apex of the canopy, and width, considering the average of two measurements from the edge of the canopy. The length (CL, cm), width (CW, cm) and perimeter (CP, cm) of the cladodes were measured, and the number of cladodes (NC, units) determined by counting the cladodes in order of appearance on the plant (i.e., first-order, second-order, third-order and so on). The total number of cladodes was determined summing the cladodes by order.

The cladode area (CA) and CAI were determined from the morphometric data as per the equations proposed by Silva et al. [87] and Pinheiro et al. [83] (Equations (11) and (12)). In addition, from the dry mass yield and cladode measurements, we calculated the morphophysiological indices and phenology using a sigmoidal model with three parameters (Equation (13)) and accumulated degree-days (Equation (14)) [61,88].

(11) $$\mathrm{CA}=1.6691\;\times \left(\frac{1\;-{\;e}^{\left(-0.0243\;\times \;\mathrm{CP}\right)}}{-0.0243}\right)$$

(12) $$\mathrm{CAI}=[\overset{i=1}{\underset{n}{\sum }}\left(\mathrm{CA}\right)/\frac{10,000}{\left(S1\;\times \;S2\right)}\;]$$

(13) $$\;y=\frac{a}{{1+e}^{\left(-\;\frac{x-x0}{b}\right)}}$$

(14) $$\mathrm{ADD}=\overset{n}{\underset{j=1}{\sum }}[\frac{\left(T_{max}{+T}_{min}\right)}{2}\;-\;\mathrm{Tb}]$$

where *i* is the observation number, *n* is the total number of observations, 10,000 is the conversion factor from cm^2^ to m^2^ and S1 × S2 is the spacing between the rows and plants (1.0 × 0.2 m), respectively.

The following parameters were used in the morphophysiological and phenological analysis: *y* is the response variable (e.g., cladode dry matter, cladode area index and the number of cladodes); *a* is the maximum value for the growth rate (i.e., the distance between the two asymptotes); *x* is the accumulated degree-days; *x*0 is the number of degree-days necessary for the plant to express 50% of the maximum growth rate (i.e., the inflection point of the curve); *b* is the number of degree-days necessary to the start of growth; ADD is the accumulated degree-days (°Cday); *j* is the daily time step; *n* is the total number of days; *T_max_* is the daily maximum air temperature (°C); *T_min_* is the daily minimum air temperature (°C); *Tb* is the lower base temperature (°C). *Tb* is the minimum temperature at which cactus cladodes grow (22 °C) [61]. If the daily average temperature drops below 22 °C, the ADD becomes negative. Negative ADD values were set to zero.

The growth indices were then quantified: absolute growth rate (AGR, Mg ha^−1^ °Cday^−1^); relative growth rate (RGR, Mg Mg^−1^ °Cday^−1^); net assimilation rate (NAR, Mg ha^−1^ °Cday^−1^); specific cladode area (SCA, Mg^−1^ °Cday^−1^); and cladode emission rate (CER, units °Cday^−1^) based on earlier studies [61]. The cladode emission rate is a very important variable in phenological analysis, as it is used to obtain the phenological phases of the crop [60,61]. We determined the cutting time as when the absolute growth rate reached 25% of the maximum peak [61].

### 4.6. Measuring the Plant Water Status

The cladode water content (CWC, %) was calculated from the fresh matter weight (FM) and dry matter weight (DM) of the samples (Equation (15)) and from the cladode succulence (CS, g cm^−2^) (Equation (16)) [5,89].

(15) $$\mathrm{CWC}=\left(\frac{\mathrm{FM}\;-\;\mathrm{DM}}{\mathrm{FM}}\right)\times \;100\;$$

(16) $$\mathrm{CS}=\left(\frac{\mathrm{FM}\;-\;\mathrm{DM}}{\mathrm{CA}}\right)$$

### 4.7. Indicators of Water and Environmental Stress

In this study, we examined the Normalised Difference Vegetation Index (NDVI) and the Evaporative Stress Index (ESI) for seasonal and interannual variations. The association between the NDVI and ESI allows the health status of plants to be understood in response to the intensity of hydrological drought [90,91]. The NDVI was derived using images from the Landsat-8 Operational Land Imager (OLI) and Landsat-7 Enhanced Thematic Mapper (ETM+), available on the United States Geological Survey (USGS) website at 30 m resolution for the red (0.64–0.67 μm) and near-infrared (0.85–0.88 μm) bands [22,92]. Landsat images acquired from April 2015 to April 2017 were then processed using the Google Earth Engine (GEE) platform. With the GEE Application Programming Interface (API) we implemented code written in JavaScript to quantify the NDVI [22]. In addition, a pixel-quality attribute mask was applied to the images to mask clouds, cloud shadow and water using the CFMask algorithm, with the values expressed as surface reflectance [93,94]. The NDVI can take dimensionless values ranging from −1 to +1, being positive when the crops show photosynthetic activity, and generally referring to bodies of water when negative. On the other hand, low positive NDVI values are characteristic of stressed vegetation or vegetation with a small leaf area.

Although the ESI can be quantified via remote sensing, we used field data to quantify it as per Equation (17) [58]. The ESI generally ranges from 0 to 1 and is linked to the evaporative demand of both the surface and the atmosphere. When the ESI is close to 1, it indicates water stress in the ecosystem; when it approaches 0, it indicates the absence of water stress [41,91]. In addition, the reference evapotranspiration (ET_0_) was estimated using the FAO-56 Penman-Monteith method and then multiplied by the crop coefficient (K_c_) to obtain the potential crop evapotranspiration (PET_c_) [34].

(17) $$\mathrm{ESI}=1-\frac{\mathrm{ET}}{{\mathrm{PET}}_{c}}$$

### 4.8. Soil Moisture

We considered the soil water balance method proposed by Thornthwaite–Mather to determine the available water fraction (AWF) over time [95,96,97,98]. This fraction expresses the total available water that a crop can extract without undergoing water stress [95,97]. Daily soil and micrometeorological data from the tower located in the experimental area were used. The available water capacity of the soil and a respective root-system depth of 75 cm and 60 mm were considered as per Aparecido et al. [99] and Almagbile et al. [100].

### 4.9. Statistical Analysis

In the first step, we applied nonlinear sigmoid functions for the morphophysiological indices and cutting time, adopting the significance of the F-test (*p* < 0.05) and the coefficient of determination (R^2^ > 0.85) as the criterion for choosing the model. Data on the NDVI, ET_0_, ESI, available water fraction and rainfall were submitted to descriptive statistics and expressed as the mean and standard deviation. In the next step, the time-scale data of the energy balance components (*R_n_*, *LE*, *H* and *G*), ET and VPD for the four seasons under study (i.e., wet season, dry season, wet-dry transition and dry-wet transition) were submitted to one-way analysis of variance (ANOVA) by F-test (*p* < 0.05). The mean values were compared by Tukey’s HSD (honestly significant difference) test for multiple comparisons (*p* < 0.05). Data on the cladode water content and environmental conditions were evaluated using boxplots showing the median, interquartile range and 1.5 times the interquartile range.

Finally, the interrelationships between the plant parameters and environmental conditions were tested using principal component analysis (PCA). We applied PCA to the mean value of the environmental variables (*R_n_*, *LE*, *H*, *G*, VPD, ESI, NDVI and ET_0_) and plants variables (ET, NUE, WUE, AGR, RGR, NAR, SCA, CWC, CS, RUE and yield). The response variables were standardised using the z-transform (mean = 0, standard deviation = 1), subtracting from the mean value and then dividing by the standard deviation. This is due to the different magnitudes and units of the variables under study and makes them directly comparable. Significant principal components were selected according to the Kaiser criterion, considering only eigenvalues greater than 1.0 [1,22,56]. The analysis was carried out using the R software [101].

## 5. Conclusions

In conclusion, our findings demonstrate that the energy budget showed seasonality, with the latent heat flux (*LE*) and sensible heat flux (*H*) compromised more strongly during the dry-wet transition season. The values for the *H*/*R_n_* ratio on a seasonal scale were always higher than those of the *LE*/*R_n_* ratio during each of the four seasons. In general, during the wet and dry seasons, the cactus vegetation maintained high evapotranspiration (1.08 mm day^−1^). The dry season showed the lowest net radiation (5.89 MJ m^−2^ day^−1^). The soil heat flux (*G*) was strongly dependent on the rainfall and the end of the wet season; there is no difference in soil heat transfer over the seasons. The seasonal fluctuations in the NDVI and growth rates confirm the influence of the wet and dry seasons. However, the cactus plants maintained their growth even during the most critical periods. When there is moisture in the soil and a low vapour pressure deficit, more energy is consumed for *LE*, resulting in greater evapotranspiration; while there is a water restriction, more energy is attributed to *G* and *H*, also increasing the Bowen ratio and Evaporative Stress Index. We found that the energy distribution is greater and preferably converted into sensible heat. The study also demonstrated how principal component analysis can help and be effectively used to understand the way plants respond to environmental factors, and which variables of the energy balance are correlated with plant growth.

Moreover, our results can be a particularly valuable baseline for studies on cacti in semi-arid ecosystems, where we believe this to be a pioneering study, with application of the surface energy balance and flux partitioning to *Nopalea cochenillifera* (L.) Salm-Dyck. Finally, these findings may also be useful for decision makers in environmental management, the rehabilitation of degraded lands, and climate change, targeting CAM plants as an alternative way of reducing heat fluxes, and for their tolerance to low water availability. In future studies, it would be interesting to quantify CO_2_ fluxes in the soil and in the environment in wetlands and drylands cultivated with cactus.

## Figures and Tables

**Table 1:** Energy budget component partitioning during the wet, wet-dry, dry and dry-wet seasons in an area of cactus.

Season	Partition Ratio (%)		
	*LE*/*R_n_*	*H*/*R_n_*	*G*/*R_n_*
Wet	30	57	13
Wet-dry	23	58	19
Dry	26	55	19
Dry-wet	17	64	19
Mean ratio	24	58.5	17.5

*R_n_* is the net radiation; *LE* is the latent heat flux; *H* is the sensible heat flux; *G* is the soil heat flux.

**Table 2:** Summary of the efficiency of the biophysical parameters, Bowen ratio and yield in the cactus at the end of the experimental period.

Variable	Mean Value	Standard Deviation
*β* (dimensionless)	3.53	±2.45
Yield (Mg ha^−1^)	12.47	±2.20
RUE (g MJ^−1^)	3.95	±0.70
WUE (kg m^−3^)	1.75	±0.31
NUE_[Calcium]_ (mg m^−3^)	29.03	±1.14
NUE_[Magnesium]_ (mg m^−3^)	12.44	±2.25
NUE_[Phosphorus]_ (mg m^−3^)	62.13	±11.71
NUE_[Potassium]_ (mg m^−3^)	50.88	±5.50
NUE_[Sodium]_ (mg m^−3^)	0.22	±0.02

RUE is the radiation use efficiency; *β* is the annual Bowen ratio based on annual energy budgets; WUE is the water use efficiency; NUE is the nutrient use efficiency; Yield is the yield of aboveground dry biomass.

**Table 3:** Consistency criteria for data generated by the Bowen ratio method under non-advective conditions.

Available Energy ^†^	Vapour Pressure Gradient	Bowen Ratio	Heat Flux
*R_n_* − *G* > 0	∆*e* > 0	*β* > −1	*LE* > 0 and *H* ≤ 0 for −1 < *β* ≤ 0 or *H* > 0 for *β* > 0
	∆*e* < 0	*β* < −1	*LE* < 0 and *H* > 0
*R_n_* − *G* < 0	∆*e* > 0	*β* > −1	*LE* > 0 and *H* < 0
	∆*e* < 0	*β* < −1	*LE* < 0 and *H* ≥ 0 for −1 < *β* ≤ 0 or *H* < 0 for *β* > 0

^†^ data considered satisfactory as described by Perez et al. [77]. *R_n_* is the net radiation, *G* is the soil heat flux, ∆*e* is the difference in vapour pressure between the two measurement heights, *β* is the Bowen ratio, *LE* is the latent heat flux and *H* is the sensible heat flux.

**Table 4:** Summary of error types when faults occur using the Bowen ratio-energy balance method.

Type of Error	Applied Conditions
*A*	*R_n_* − *G* > 0, ∆*e* > 0 and *β* < −1 + |*ε*|
*B*	*R_n_* − *G* > 0, ∆*e* < 0 and *β* > −1 − |*ε*|
*C*	*R_n_* − *G* < 0, ∆*e* > 0 and *β* > −1 − |*ε*|
*D*	*R_n_* − *G* < 0, ∆*e* < 0 and *β* < −1 + |*ε*|
*E*	Referring to a rapid change in temperature and vapour pressure.

*β* is the Bowen ratio, and *ε* is the error interval that defines the threshold for excluding values of the Bowen ratio close to −1 Perez et al. [77].

## Data Availability

The data presented in this study are available on request from the corresponding author.
